# Hijackers, hitchhikers, or co-drivers? The mysteries of mobilizable genetic elements

**DOI:** 10.1371/journal.pbio.3002796

**Published:** 2024-08-29

**Authors:** Manuel Ares-Arroyo, Charles Coluzzi, Jorge A. Moura de Sousa, Eduardo P. C. Rocha

**Affiliations:** Institut Pasteur, Université de Paris Cité, CNRS UMR3525, Microbial Evolutionary Genomics, Paris, France

## Abstract

Mobile genetic elements shape microbial gene repertoires and populations. Recent results reveal that many, possibly most, microbial mobile genetic elements require helpers to transfer between genomes, which we refer to as Hitcher Genetic Elements (hitchers or HGEs). They may be a large fraction of pathogenicity and resistance genomic islands, whose mechanisms of transfer have remained enigmatic for decades. Together with their helper elements and their bacterial hosts, hitchers form tripartite networks of interactions that evolve rapidly within a parasitism–mutualism continuum. In this emerging view of microbial genomes as communities of mobile genetic elements many questions arise. Which elements are being moved, by whom, and how? How often are hitchers costly hyper-parasites or beneficial mutualists? What is the evolutionary origin of hitchers? Are there key advantages associated with hitchers’ lifestyle that justify their unexpected abundance? And why are hitchers systematically smaller than their helpers? In this essay, we start answering these questions and point ways ahead for understanding the principles, origin, mechanisms, and impact of hitchers in bacterial ecology and evolution.

## Abstract 2

Microbial evolution is shaped by mobile genetic elements, but recent work shows that many of these depend in turn on other mobile genetic elements. These chains of functional dependencies raise numerous questions in terms of genetics, molecular biology, ecology and co-evolutionary processes.

## Introduction

The past few decades have transformed our understanding of microbial evolution. It is now clear that there are vast intra-species variations in the gene repertoires of microbial populations and a strong suspicion that such differences explain local adaptation. This diversity leads to large species pangenomes, often much larger than the average individual genome, and to closely related strains differing markedly in gene number and type [1,2]. These variations are caused by processes of gene gain via horizontal gene transfer driven by mobile genetic elements (MGEs, Box 1) [3]. The latter encode core functions required for their horizontal and/or vertical mobility, as well as accessory functions that favor the MGE by increasing its host’s growth or survival.

**Box 1. Glossary**

**Conjugation**

A molecular process allowing the transfer of (usually single stranded) DNA between cells using a mating pair formation system (such as a conjugative pilus), a relaxase and an origin of transfer.

**Conjugative elements**

MGEs capable of autonomous conjugation (conjugative plasmids or ICEs).

**Helper MGE**

MGE that can mobilize another MGE.

**Integrative conjugative elements (ICEs)**

MGEs that integrate the chromosome and encode all major components for conjugation.

**Integrative mobilizable elements (IMEs)**

MGEs that integrate the chromosome and encode an origin of transfer with (iMOB) or without (iOriT) a relaxase, but lack the mating pair formation system.

**Mobile genetic element (MGE)**

Genetic elements that encode enzymes and other proteins mediating the mobility of DNA within genomes or between bacterial cells.

**Mobilizable plasmids**

Plasmids that are unable to move between cells independently but can be mobilized by conjugation when the protein machinery is encoded in part (pMOB) or completely (pOriT) in another MGE.

**Origin of transfer (*oriT*)**

The origin of transfer by conjugation is a small DNA motif that is recognized by the relaxase in the beginning of the process of conjugation.

**Phage satellites**

MGEs that are not phages but encode molecular mechanisms or DNA signals favoring their packaging in viral particles totally or partially produced by phages.

**Prophage**

A phage that integrates into the host genome during a process called lysogeny.

**Transduction**

Process whereby a viral particle transfers into another bacterium DNA that does not encode the phage particle (bacterial, satellite).

**Transposable element**

MGE able to change its position within and between DNA molecules.

MGEs often impose a fitness cost to the bacterium host because they disrupt gene expression, impair growth, and can even cause its death. [4–6]. These deleterious effects to the host may or may not be compensated by MGE-encoded accessory traits such as antibiotic resistance, immune defense, or virulence. Hence, the interests of MGEs and their hosts are sometimes aligned and other times misaligned, resulting in a shifting balance in the parasitism–mutualism continuum [7,8]. There is a rich literature on these wavering interactions between single MGEs and their hosts. Yet, recent results strongly suggest that such interactions can only be fully understood on a broader context because MGEs are rarely alone within cells.

Some MGEs transfer autonomously between cells: bacteriophages (or phages) are viruses that infect bacteria and sometimes transfer bacterial DNA between hosts [9], whereas conjugative elements are capable of transferring (usually single-stranded DNA) between cells via a process called conjugation (Box 1). Other MGEs that we will refer to as Hitcher Genetic Elements (hitchers or HGEs) cannot transfer autonomously and must use functions of autonomous helper MGEs to transfer (Box 1). Importantly, the term “helper” does not necessarily imply altruism. It merely describes an MGE that is involved in the mobility of a hitcher. As we will describe below, some hitchers require other hitchers for their mobilization (beyond also strictly requiring a helper). For simplicity, we will name both types of hitchers in the same way, since both types of elements depend on a helper.

Recent works have shown that hitchers are very abundant and may be key to understand the mobility of many bacterial genes [10,11]. The view of the interactions between bacterial genomes and MGEs is thus evolving. If traditionally one would think of pairwise interactions between a bacterium and its MGE, the focus is now shifting towards MGEs and their hosts forming a network of complex functional interactions, ranging from antagonism to mutualism. This brings to the fore major unsolved mysteries. First, it highlights the need to unravel the mechanisms of interactions shaping the mobilization of MGEs and, ultimately, understand who moves whom and by which mechanisms. Second, while it is common to regard HGEs as hyper-parasites, it is unclear what are their actual costs, and whether these could be offset by the advantages they provide to both helpers and the bacterial host. Third, there is little data on the evolution of the mechanisms of co-mobilization leading to the emergence of HGEs. Finally, a better understanding of hitchers’ emergence, interactions, costs, and benefits could explain why they evolved to be mobilizable and not autonomously mobile. Here, we put forward the current knowledge and many questions surrounding hitchers with the goal of sketching the way ahead.

## Who are the known hitchers, and who’s helping them move?

Phages encode genes to replicate their DNA, to produce, assemble and package the viral particle containing the DNA, and to lyse the bacterial cell to release the newly formed viral particles in the environment, from where they can infect new bacterial hosts. Virulent phages nearly immediately replicate in the host cell resulting in cell death (lytic cycle), while temperate phages can follow the same path or a lysogenic cycle, whereby the phage integrates into the host genome and replicates with it. The temperate phage DNA either integrates into the bacterial chromosome (as a prophage) or remains episomal (as a phage-plasmid) being vertically transmitted until it re-enters the lytic cycle and eventually kills the host cell (Box 1). Sometimes bacterial DNA is packaged as it if was phage DNA in the viral particles, where it can be transferred to other bacterial cells by transduction (Box 1).

**Fig 1:**
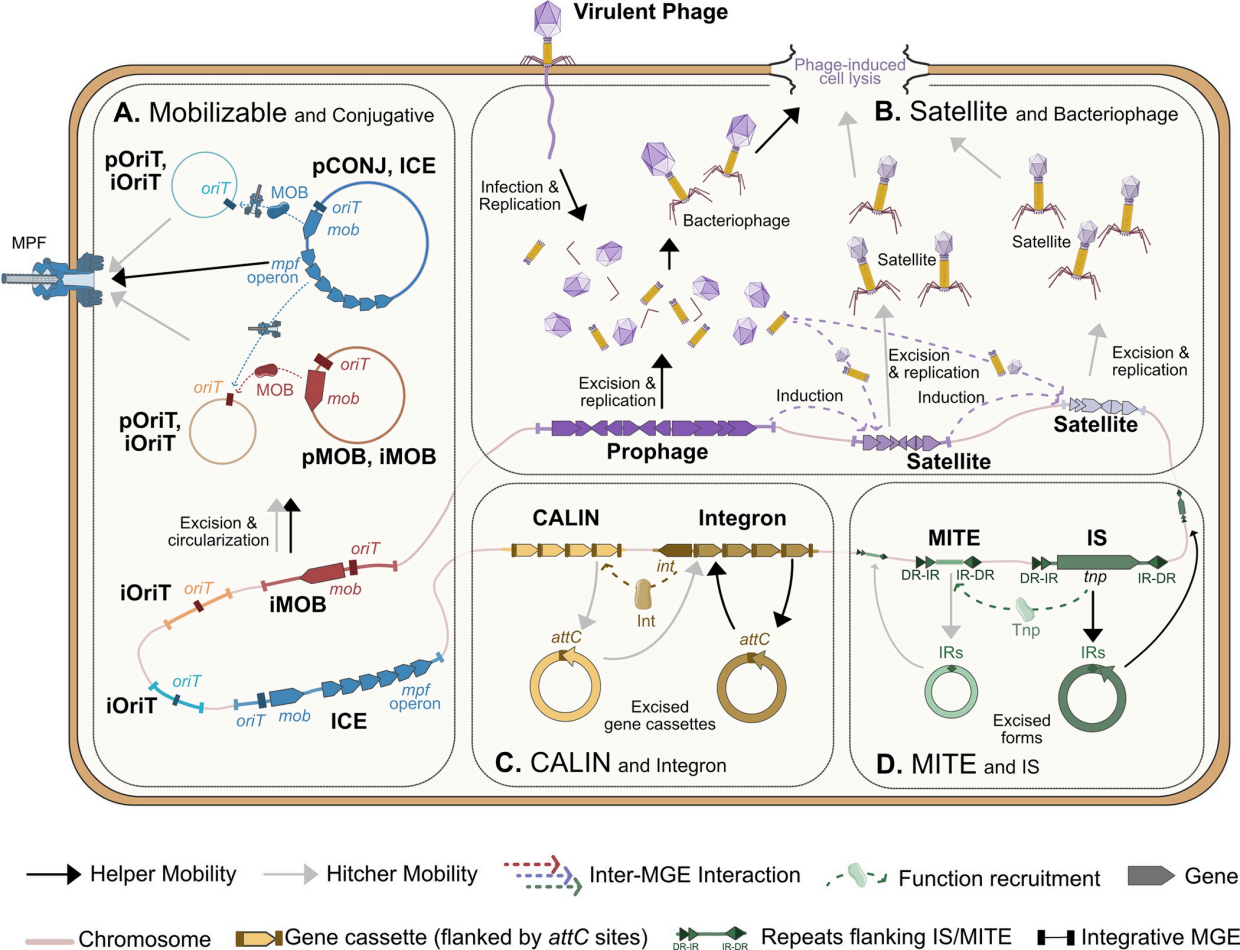
The diversity of hitchers and helper elements, and how they interact within the cell. The black continuous arrows indicate the mobility of the helper MGE, whereas the gray continuous arrows represent the hitcher mobility. Colored dashed arrows indicate interactions between different types of MGE. (**A**) The helper pCONJ assembles the MPF system for its own mobility. Mobilizable plasmids (pMOB) encode for their own relaxase (MOB) and *oriT*, sufficient to be mobilized by the MPF encoded by a pCONJ. pOriTs carry their own *oriT* but require a MOB encoded by a pCONJ (above) or a pMOB (below). Before conjugation, ICEs and IMEs need to be excised and circularized. The excised forms show similar mechanism to plasmids. ICEs assemble the MPF system for their own transfer. Some IMEs, such as iMOB, encode their own relaxase and *oriT* and can therefore use the ICE’s MPF. On the other hand, iOriTs only carry their own *oriT* and need the MOB encoded by an ICE (bottom) or by an iMOB (above), to be mobilized by the MPF. Some iMOBs and iOriTs can rely on the MOB and MPF system encoded in pMOBs and pCONJ, whereas some pMOBs and poriTs can rely on iMOBs and ICEs. (**B**) Bacteriophages produce all the elements required for the assembly of viral capsids, the genome packaging, and the cell lysis. Phage induction may induce satellites, which use resources provided by the phage (e.g., left satellite). Likewise, the induction of some satellites may trigger the induction of additional satellites and their mobility (e.g., right satellite). Some satellites shrink the capsids of phages, whereas others produce their own smaller capsids, which results in phage particles carrying only the satellite DNA in small capsids. (**C**) Integrons use their own integrase (Int) enzymes to shuffle their gene cassettes array. Clusters of *attC* sites lacking integron-integrases (CALINs) are cassette arrays that lack an integrase and depend on an integron’s integrase to be mobilized. (**D**) IS encode a transposase (Tnp) enzyme that mediates their intragenomic mobility. Only one of many different mechanisms of transposition is shown. MITEs are hitchers that utilize the transposase of their helpers to be mobilized within the genome. DR, direct repeat; ICE, integrative conjugative element; IME, integrative mobilizable element; IR, inverted repeat; IS, insertion sequences; MGE, mobile genetic element; MITE, miniature inverted repeat transposable element; MPF, mating pair formation; pCONJ, conjugative plasmid.

Phage satellites (Box 1) are a diverse group of HGEs that specialized in packaging their genome, instead of the one of the helper phage, in viral particles encoded by the latter and hijacked by the satellite (Fig 1B). The first described phage satellite, P4, was the only one known for decades, and is now the reference of a characteristic family of satellites known as P4-like [12]. In the last few years, other families of phage satellites have been uncovered, such as the phage inducible chromosomal islands (PICI) [13,14], capsid-forming PICI (cfPICI) [15], PICI-like elements (PLE) [16], and phage-inducible chromosomal minimalist islands (PICMI) [17]. Additional families of phage satellites in marine (VEIMEs [18] and Tycheposons [19]) or soil bacteria [20] have been recently proposed. Some elements are satellites of single-stranded DNA phages, e.g., the plasmid pDolos [21]. Phage satellites depend on helper phages to produce all or parts of the viral particle, or even to replicate the satellites’ DNA. They can hijack viral particles by manipulating the capsid size (e.g., [22]) or by redirecting packaging towards their own DNA (e.g., [23]). Some satellites, like the *Staphylococcus aureus* satellite SaPI3, add an extra layer of complexity to the chain of functional dependencies between MGEs because they require another satellite for induction and cannot be induced directly by the helper [24]. This suggests that satellites are involved in a complex hierarchy of functional dependencies within the bacterial cell (Fig 1B).

Autonomous conjugative elements transfer copies of their genomes into neighboring recipient cells using their own conjugation system that is costly but does not usually entail donor cell death [25,26]. Conjugative plasmids (pCONJ) are extrachromosomal fragments of DNA that replicate independently of the bacterial chromosome. They encode a relaxase that initiates the transfer of plasmid DNA at their origin of transfer (*oriT*) and a mating pair formation (MPF) system that connects the donor and recipient cells and serves as channel for the plasmid transfer. Integrative conjugative elements (ICEs) (Box 1) are also autonomously transferred by conjugation, but encode an integration and excision module to integrate into the bacterial chromosome, thereby replicating with it.

Some MGEs do not encode a functional conjugative apparatus and thus require those encoded by autonomous conjugative elements to transfer horizontally. These conjugative HGEs may be plasmids that encode a relaxase and an *oriT* (pMOB), plasmids that carry only an *oriT* (pOriT), or integrative mobilizable elements (IMEs) carrying an *oriT* with a relaxase (iMOB) or without it (iOriT) (Box 1). Since the conjugation machinery of ICEs and plasmids is homologous [27], pMOBs, pOriTs, and IMEs can be potentially mobilized by both types of conjugative elements [28–30] (Fig 1A and 1C). Conjugative HGEs may use proteins from multiple elements, some of which may also be hitchers themselves, thereby establishing a hierarchy of interactions within the cell that is necessary for their transfer [31]. Such plasmids are common: a third of those having only an *oriT* transfer between cells using a relaxase from one plasmid and a conjugative system from another [10].

The focus of this text is on co-mobilization in horizontal gene transfer. Yet, while most known mobilizable elements are horizontally transferred between cells, relations of functional dependency are also observed for MGEs involved in intra-genomic mobility. The best described case concerns the association between transposable elements (Box 1) and miniature inverted repeat transposable elements (MITEs), which are encoded by more than 50% of bacteria [32] (Box 1). These short elements (ca. 300 nt) lack protein coding genes and are transposed by transposases encoded in insertion sequences or other MGEs [33]. They can thus be considered HGEs of transposable elements (Fig 1D).

Integrons are elements encoding a specific integrase that mediates recombination between *attC* sites flanking gene cassettes [34]. This mechanism results in the integration of novel cassettes and shuffling of old ones. While not strictly speaking an MGE, integrons can exchange cassettes with other integrons and thus participate in intra-genomic genetic mobility. Cassette arrays lacking an integrase are called CALIN [35], can be mobilized in trans by complete integrons, and may thus also be thought of as HGEs of integrons (Fig 1C).

The interactions between agents of intra-genomic mobility are important for the MGEs involved directly on inter-genomic mobility (horizontal transfer). This is especially true for conjugative HGEs and their helpers, which contain numerous transposable elements, MITEs, integrons, and CALINs [36,37]. These elements are key for the transfer of other genes between plasmids and/or integrative MGEs, e.g., for exchanges of antibiotic resistance genes between conjugative and mobilizable plasmids [38]. While they do not allow horizontal transfer, they lead to novel assemblies of potentially adaptive functions in the helpers and hitchers transferring between bacteria.

The ways HGEs attain helper-mediated genetic mobility differ widely. Some hitchers encode sophisticated mechanisms to actively subvert their helpers (viral particles or conjugative apparatus). This is common in phage satellites, many of which encode genes dedicated to physically redirect the packaging of viral particles towards the satellite genome [23], to reshape or exclude the viral capsid to fit the satellite DNA [22], and/or exclude the packaging of helper phage genomes in viral particles [39]. HGEs mobilized by conjugation often encode their own relaxases and coupling proteins to better interact with the type IV secretion system of the conjugative element [40,41]. Such interactions do not necessarily preclude the use of the conjugative system by the helper, but sometimes they do. The IME SGI1 encodes a protein (TraGs) that reshapes the pilus of its helper conjugative plasmid to enhance its transfer at the expense of the latter [42]. These are sophisticated molecular mechanisms of subversion that require proteins encoded by the hitcher.

Other hitchers use more passive mechanisms of subversion and depend only on the presence of DNA motifs in their genome that are recognized by the helpers’ transfer machinery. For example, the mobilization of PICMI satellites seem to depend only on the presence of a DNA motif directing PICMI packaging to viral particles [17], and many plasmids only encode an origin of transfer to be mobilized by conjugative plasmids [10]. This is also the case of MITEs, which lack protein coding genes and whose mobilization relies solely in the recognition of their DNA motifs by transposases encoded in helper transposable elements. As we will discuss later, the differences between these mechanisms of subversion may impact the fitness of their helpers.

## How many hitchers?

Prokaryotic genomes have plenty of MGEs. More than half encode prophages, transposable elements, MITEs, and nearly as many have plasmids. Phages and conjugative elements have been widely studied but might be a relatively small fraction of the MGEs present in genomes. The search for genes coding the key mechanisms of plasmid mobility revealed that only 25% are autonomously conjugative [43,44] and 7% are phages [45]. Hence, more than two thirds of the plasmids are either mobilizable or non-transmissible. Recent studies in the model species *E*. *coli* and *S*. *aureus* strongly suggest that most of these plasmids are HGEs mobilized by autonomous conjugative elements [10,46]. Some of these plasmids could also be passively mobilized by phage transduction [47], especially those in high copy number [48]. The analysis of MGEs integrating bacterial chromosomes is more difficult because delimiting poorly known elements is challenging. Yet, bacterial chromosomes have a small number of regions (“hotspots”) where most genetic exchanges with other bacteria take place, partly because they are targeted by the site-specific recombinases of MGEs [49]. While hotspots accumulate the majority of phages and ICEs [50], many encode neither of them and instead have MGEs that have been named by their phenotypic effect as a genomic island (e.g., pathogenicity islands, resistance islands, symbiosis islands) [49].

Many of these genomic islands are now being revealed as phage satellites or IMEs. Until recently, only a handful of satellites had been identified. In just 1 year, 2 new families were discovered and characterized in detail [15,17], 3 additional novel types of satellites were uncovered in Cyanobacteria and Actinobacteria [18–20], a phage-plasmid-satellite was described [21] and several thousands of novel satellites of these families were identified in bacterial genomes [11]. Likewise, novel IMEs are regularly uncovered across various Phyla, even if they have diverse names such as genomic islands (e.g., SGI1 mobilized by IncA/C plasmids) [51], transposons (e.g., Tn*4451* mobilized by the plasmid RP4) [52], or nonreplicating bacteroides units (e.g., NBU1 mobilized by the plasmid R751 or the ICE Tcr-ERL) [53]. ICEs are more numerous than conjugative plasmids among sequenced genomes [27], so one can expect that a lot of IME diversity remains to be explored. These hitchers may account for a large fraction of the genomic islands whose mechanisms of mobility have remained unknown for decades [54–56].

**Fig 2:**
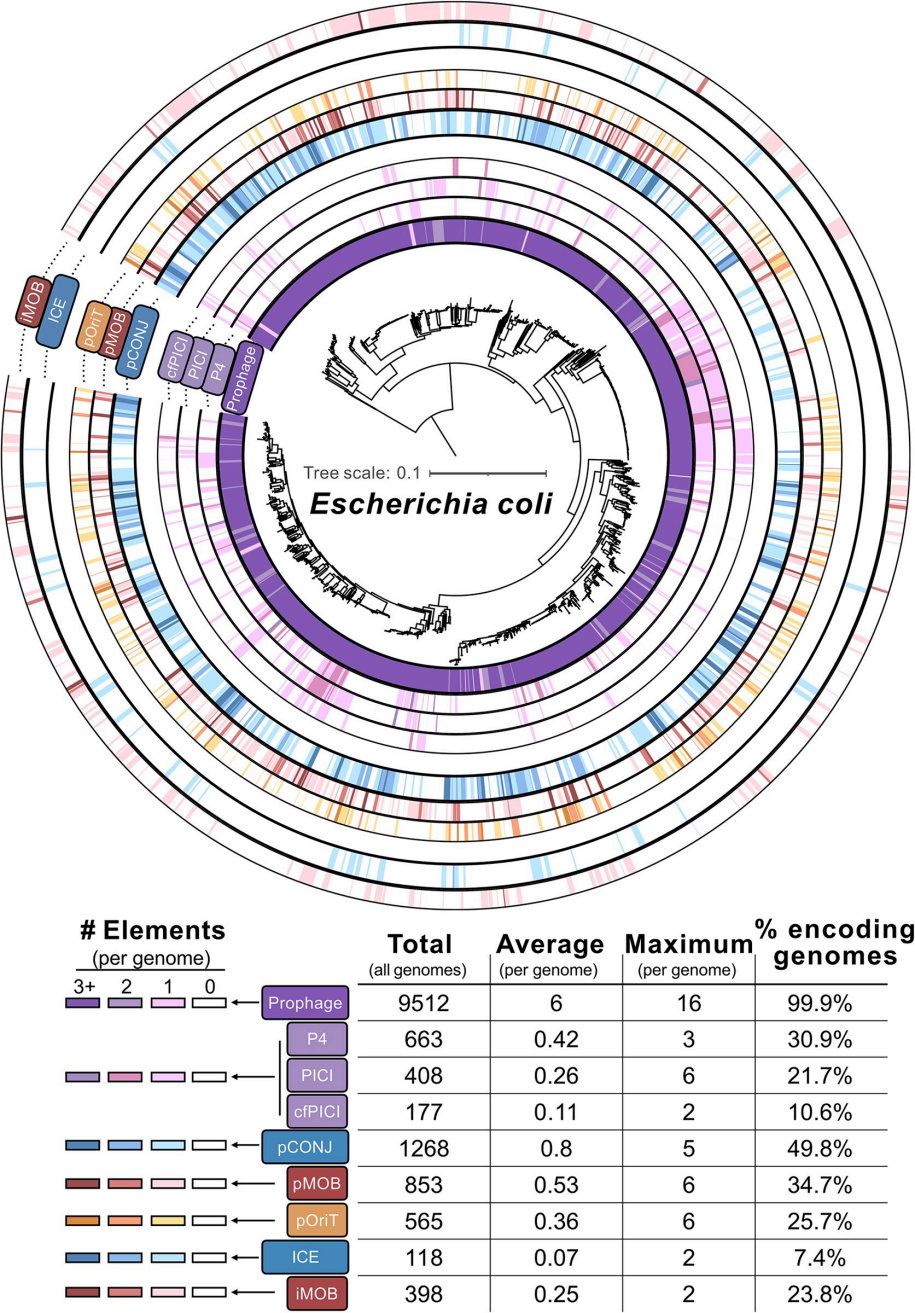
Distribution of hitchers and their helpers in *E*. *coli* genomes. Phylogenetic tree built from the core genes of 1,585 *E*. *coli* complete genomes retrieved from RefSeq (May 2021). The tree is annotated for the presence of prophages (detected with VirSorter2) [59], phage satellites P4, PICI and cfPICI (detected with SatelliteFinder) [11], conjugative plasmids, ICEs, and conjugative-based HGEs (identified with CONJScan v.2) [60]. In each circle, darker shades correspond to higher numbers of the respective elements in the corresponding genomes. The legend is at the bottom of the figure, to the left of a table with the total number of MGEs, average per genome, maximum per genome and the percentage of genomes harboring the MGE. cfPICI, capsid-forming PICI; ICE, integrative conjugative element; MGE, mobile genetic element; PICI, phage-inducible chromosomal islands.

Knowledge about HGEs remains concentrated in a limited number of elements from a few bacterial species, notably in *E*. *coli*. Most genomes of this species harbor at least 1 prophage (and up to 16) and 43% have at least 1 satellite (Fig 2). Around 52% of the genomes carry between 1 and 5 fully autonomous conjugative elements and 58% carry at least 1 conjugative hitcher. Hitchers can be numerous in individual bacteria, with some genomes carrying up to 6 phage satellites and others carrying 10 elements mobilizable by conjugation. Other genomes have multiple mobilizable elements of each type, e.g., a single genome (*E*. *coli* O157:H7 strain USDA5905) has 3 phage satellites, 5 mobilizable plasmids, and 1 iMOB. Despite the abundance of hitchers in certain bacteria, the frequency of HGEs may vary widely between closely related genomes. This scattered distribution across the phylogenetic tree of the species can be explained by frequent events of horizontal transfer and loss of hitchers. For plasmids, hitchers may be lost by mutations in replication initiators or segregation systems [57]. For the other elements, rounds of gene deletions may fully delete the elements. Recombination with exogenous homologous DNA, arriving by transformation in naturally transformable species or by the action of other MGEs (transduction, conjugation) in the others, can also “cure” bacterial genomes from integrative MGEs [58]. Finally, many MGEs are costly to the bacterial host and they may disappear because their host is purged by natural selection. The outcome is a patchy distribution of elements across a species, most of which have been recently acquired.

*E*. *coli* is among the best studied bacteria in relation to HGEs, even if some may remain unidentified, and has many such elements. It is likely that other species also harbor many hitchers. But how can we find them? Novel elements resembling known HGEs can often be identified by sequence similarity, as above. For example, PICIs were detected in 35% of *Staphylococcus* genomes [11], 34% of *S*. *aureus* genomes carry known pOriTs [10], and a fourth of all bacterial plasmids have a relaxase and are thus mobilizable by conjugation [43]. The true difficulty lies in identifying elements that differ from the known HGEs. One approach is to study the mechanisms of mobilization of helpers and find other MGEs by homology. The presence of some genes or DNA motifs might be sufficient to identify potential mechanisms of mobilization, e.g., the presence of genes encoding relaxases and *oriTs* suggest that the hitcher is mobilized by conjugation and the presence of genes encoding terminases or *cos*/*pac* sites suggest the element is mobilized by phages. Yet, some conjugative elements and phages are poorly known, some HGEs lack protein coding genes homologous to the helper, and DNA motifs such as *oriTs* or *cos/pac* are often unknown or hard to identify [61]. It is also likely that the mobilization of completely novel HGEs uses unknown mechanisms. Their discovery will require the integration of experimental and computational approaches to analyze mobilized DNA in bulk, e.g., by identifying satellites packaged in viral particles through sequencing of phage lysates [18].

## Who moves whom?

Now that thousands of HGEs are being unraveled, one must identify their helpers to infer the networks of interactions between mobile genetic elements within cells. The co-occurrence between helpers and HGEs could provide clues to their transfer compatibility, i.e., the ability of a given helper to mobilize a given hitcher. Yet, as shown above (Fig 2), genomes may have multiple hitchers and helpers, and there is no guarantee that they are transfer-compatible. For example, phages HK106 and HK446 were found to mobilize the satellite cfPICI EcClEDL933, but neither is native to the *E*. *coli* strain where the satellite was discovered. Strikingly, this strain encodes 17 prophages, none of which mobilized the satellite at detectable frequencies [15]. Inversely, some genomes have hitchers without having any possible helper. In the *E*. *coli* analysis described above, 24% of the genomes have hitchers for conjugative elements but not the conjugative elements themselves. Hence, co-occurrence has not yet been shown to allow systematic matching of hitchers with helpers. In some cases, the elements are expected to co-occur rarely if ever. This is the case of hitchers whose helpers are virulent phages [17], and thus will not be stable in a cell. It is also the case of hitchers that block their helpers since in this case only one of the elements will transfer.

Another approach to find pairs of compatible helpers and hitchers is to search for homologous DNA motifs that are present in the genomes of both elements, such as *oriTs* for conjugation or *cos/pac* sites for packaging in viral particles. The similarity between hitchers and helpers at these DNA motifs is essential for the former to be mobilized by the latter. Hence, pairs of elements with identical sequences should make a pair of compatible hitchers/helpers. Unfortunately, as mentioned above, these DNA sequences are often small, degenerate, or unknown.

Finally, co-integration of helpers and hitchers suggests the 2 elements are compatible. Some conjugative hitchers can transfer between cells by conduction, i.e., by co-integrating the genome of the helper in the donor cell and excising once they are in the recipient cell. In this case, the hitcher does not need to encode any function related to conjugation, not even an origin of transfer, since it is now part of the conjugative helper and transfers with it, in a way resembling insertion sequences that translocate into a conjugative plasmid. Examples of this mechanism include the co-integration of pSC101 and the conjugative plasmid R1-19, or of pML21 and R64-11 [62]. Of note, in these cases conduction is an active mechanism, i.e., the hitcher encodes genes that seem to have evolved to integrate and then excise the other element. In many cases, it may be difficult to distinguish this mechanism from casual co-integration between MGEs, e.g., driven by transposable elements.

In theory, phage satellites could also integrate conjugative elements and transfer by conduction, but satellites known so far rarely integrate conjugative plasmids [11]. Conduction is expected to be rare or inexistent between phages and satellites, because their co-integration creates a larger genome that can only be packaged in the original phage particle if the satellite is very small. A different process is sometimes observed in phages and satellites (and even in conjugative elements): some HGEs integrate their helpers after transfer. For example, some satellites were observed to integrate within prophages [63] and some IMEs within ICEs [30]. These processes are different from conduction because the co-integrate is split before transfer and the 2 elements are transferred independently. Nevertheless, they provide information on the compatibility of helpers and hitchers, since available data suggests that co-integration is used by the HGE to take control of the transmission of its helper [30].

## What are the host-ranges and helper-ranges of hitchers?

There are 2 types of host ranges for MGEs: the set of bacterial hosts they can infect and the set of bacterial hosts from where they can further transfer. The first is often broader than the latter because MGEs may be able to transfer their DNA to a host, but cannot further transfer from there. For example, some conjugative elements can transfer into eukaryotic cells, but cannot further transfer because the cell envelope is too different to allow assembly of the conjugative system [64]. Both ranges are important for the horizontal transfer of genetic information. The range of bacteria that can be infected by helper MGEs is extremely diverse because of the variations in the molecular mechanisms of transfer, integration, and replication of MGEs and because of the differences in bacterial physiology and immune defenses. Nevertheless, the range of transfer of conjugative elements and phages is quite different. Phages interact specifically with cell receptors and tend to have relatively narrow host ranges, often limited to a clade within a species or genus [65]. In contrast, the molecular interactions of conjugative elements with the recipient cell tend to be either less specific or more tolerant to the absence of specific receptors [66], and this results in broader host ranges [65].

What about HGEs? The study of these elements requires a further extension of the traditional concepts of host range because the hitcher depends on the bacterial host and on the helper. A simple prediction is that these additional constraints would further narrow the host range of HGEs. The available data suggests a more complex and intriguing picture.

One might think that if a hitcher is mobilized by a single helper, then it should have approximately the same bacterial host range. This seems to be the case with the satellite PLE: it is known to exploit only the virulent phage ICP1 [16], whose only known host is *Vibrio cholerae* [67]. As a result, this hitcher is only found within the triplet PLE-ICP1-*Vibrio cholerae*. Yet, hitchers and helpers may have different host ranges if their ability to function in the new host differs (e.g., if only one has a functioning replication initiator) or if the novel host defense systems target one element and not the other (e.g., small MGEs escape restriction more easily [68]).

Some hitchers can be mobilized by a wide diversity of helpers and this may expand their host range. For example, P4 can be mobilized by at least 5 different P2-like phages [69]. The range of helpers used by elements mobilizable by conjugation can be even broader. Relaxases of mobilizable elements (e.g., MOB_Q1_ or MOB_P5_) interact with multiple types of conjugative systems [70]. Moreover, some mobilizable plasmids carry multiple *oriTs* (e.g., pEC156) [71], or encode coupling proteins (e.g., CloDF13) [40] that expand the range of helpers that can mobilize them. The ability of HGEs to be mobilized by different helpers may dramatically increase their bacterial host range both in terms of infection (as they can hijack helpers that infect different hosts) and their ability to transfer from the novel recipient cell (since the likelihood of coinciding there with a helper for subsequent transfer is higher). Indeed, many of the plasmids with the broadest host range are mobilizable, like RSF1010/R1162 [72], pLS1 [73], or pBI143 [74]. For example, RSF1010 can be mobilized by the plasmid R388 in *Escherichia* [70], by pAtC58 in *Agrobacterium* [75], and by RP4 in the very distantly related *Mycobacterium* and *Streptomyces* [76]. Likewise, nearly identical P4-like satellites can be found in *E*. *coli* and *Klebsiella* spp. [12], corresponding to phylogenetic distances rarely crossed by phages. In theory, although this remains to be demonstrated, hitchers using different populations of helpers in distinct clades may have a key role in transferring genes across distant bacteria that their helpers, individually, cannot reach.

The helper range of some HGEs might be dynamic, since only a few mutations are required in either the hitcher or the helper to create a functional association. For example, in *S*. *aureus*, a single amino acid substitution in the relaxase of an IME allows it to recognize divergent *oriTs* [77]. Likewise, a common point mutation in the relaxase accessory gene *smpO* allows the helper conjugative plasmid pWBG749 to mobilize a broader range of mobilizable plasmids [77]. In conclusion, HGEs that can be mobilized by a large range of helpers have broader bacterial host range, and some might be just a few point mutations away from accessing a different group of helpers, and thus potentially a new set of bacterial hosts.

## The costs and benefits of hitcher mobilization: Hijackers, hitchhikers, or co-drivers?

Hitchers can be costly to the helper, to the bacterial host, or to both. In this context, the analysis of the cost of hitchers is even more complex than that of conjugative plasmids or prophages. It is usually assumed that hitcher mobilization decreases the fitness of their helpers because the hitchers compete for a common pool of resources and hijack components of the helper (e.g., conjugative pili or viral particles). This is indeed the case for some HGEs. The pMOB CloDF13 reduces the rate of transfer of its helper by competing for the conjugation machinery [78], the IME SG1 diminishes the conjugation of its helper plasmid pVCR94 [42], and PLEs abolish the helper phage reproduction [16]. The cost of hitcher mobilization to its helpers may depend on the hitcher–helper pair. For example, the cost of some PICI in terms of helper reproduction differs between helpers [39]. Other hitchers have little or no effect on the transfer of the helper. For example, the satellites cfPICI EcClEDL933 [15] and PICMI_115_ [17] had no significant effect on the production of the viral particles carrying the DNA of their helpers. Similarly, integrative elements mobilized by ICEs related to ICE*St3* [30], as well as plasmids mobilized by the conjugative plasmid pWBG749 [79], have a negligible impact in the efficiency of transfer of their helpers. Other observations further suggest that hitchers are often not costly to helpers. For example, although some conjugative plasmids encode CRISPR-Cas systems to target other plasmids, the targets are typically other conjugative plasmids and not their hitchers [80], suggesting that these mobilizable plasmids have minor impact on their helpers’ fitness.

If hitchers have little or no impact on the helper’s mobility, are there hitchers that increase the mobilization of helpers? CTXφ is a filamentous phage encoding the toxin Ctx that makes *V*. *cholerae* a deadly pathogen. Its satellite RS1 counteracts the phage repressor promoting the expression of itself and of its helper phage, resulting in increased expression of the cholera toxin gene and virulence of *V*. *cholerae* [81]. This could be a case of cooperation, where a satellite increases the transferability of its helper to improve the chances of both co-transferring into novel recipient bacteria. The regulatory networks responsible for repressing and inducing MGEs are sometimes shared by hitchers and helpers (e.g., the SgaD/C and AcaC/D protein-homologs in SGI1 IME and pVCR94X conjugative plasmid, respectively [82], or the protein E in P4 satellites and P2 phages [83]) and could allow cooperation between them or exploitation of one by the other. If helpers and hitchers can induce each other and the 2 elements respond to distinct environmental queues for induction, this might increase each other’s transferability. Yet, the benefits for a helper of an increased mobility promoted by their hitchers remains to be shown because the effects of an untimely mobilization might be counterproductive. A key question for future research is whether these genetic interactions are beneficial to the partners.

Regardless of the interactions with the helper, does HGEs mobilization carry additional costs for the bacterial host? In the tripartite relation between hitchers, helpers and the host, the costs of mobility MGE for the bacteria can be high. The horizontal transfer of helpers is almost always costly to the donor bacterium: novel phage particles usually require cell lysis for dispersion and conjugation affects growth rates in both donor and recipient bacteria [6,84]. In contrast, the mobilization of the HGE is not necessarily costly for the host. Satellites themselves have no mechanisms to lyse the cell. They require phages to transfer, and the mobilization of the latter kills the cell whether satellites are present or not. Their presence might even impart a benefit for the bacterial population in cases in which the satellite diminishes the helper reproduction. Conjugative HGEs may also incur little or no additional cost to the bacterial host because the conjugative machinery is expressed and assembled by the helper whether the hitcher is present or absent. Even if DNA transfer requires energy [85], the small size of mobilizable elements (see below) allows this cost to be kept low. Hence, the marginal cost of transfer of hitchers might be very low whenever they do not increase the transfer frequency of the helper.

## Could hitchers be hyper-mutualists?

Parasitism is defined as an antagonistic symbiotic relationship in which one partner is harmed, while the other benefits [7]. In contrast, mutualism is a symbiotic relationship in which both partners benefit, or are perceived to benefit [7]. These definitions can be applied to interactions between MGEs and the host [86]. Because transfer and carriage of MGEs have been usually assumed to be costly, they are often considered genetic parasites of bacteria [87]. Likewise, since the mobilization of HGEs may be costly to other MGEs (helpers), which may in turn be costly to the bacterial host, they could be considered as genetic hyper-parasites (i.e., parasites of the parasites) [88,89]. But is this really the case? Can hitchers be neutral, or even beneficial to both their helpers and their hosts (i.e., hyper-mutualists)? The costs and benefits of HGEs result from their mobilization (discussed above) and their stable residence in the host cell (this section).

The fate of autonomous MGEs hinges on a trade-off between their rate of horizontal transmission (usually costly to the host) and the frequency at which they are vertically inherited. The latter may be increased if the MGE carries adaptive traits [90,91]. If HGEs have reduced horizontal transmission relative to helpers, which remains to be shown, then they require improved chances of vertical transmission to persist in microbial populations. The residency of hitchers can be costly to the bacterial host even in the absence of transfer [92–94], but the co-occurrence of both hitchers and helpers may lower the cost of the former. For example, co-residence of small and large plasmids can be less costly than the sum of their individual costs, favoring their co-existence within the cells [10,95]. The mechanisms involved in these epistatic interactions remain poorly understood, but these observations show that hitchers may have little impact on hosts when the helpers are also present.

The presumably low cost of many HGEs opens the opportunity of establishing mutualistic interactions with the host. This may explain why some hitchers carry functional genes that are not directly implicated in the core functions of the MGE (mobility, replication, and/or integration) and may improve the host fitness [96]. For example, mobilizable plasmids are usual carriers of bacteriocins [97,98] and have the highest densities of antimicrobial resistance genes in *E*. *coli* and *S*. *aureus* [10]. Under bacteriocin or antibiotic pressure, bacteria carrying these HGEs will be strongly selected for. Phage satellites rarely carry antibiotic resistance genes, but some *S*. *aureus* PICIs encode virulence factors [99] and many satellites encode anti-phage defense systems [17,100,101], allowing the bacterial population to better withstand phage predation.

Co-residence of hitchers and helpers in a host provides opportunities for tripartite mutualisms on the basis that the growth and survival of the host benefits all of them. For example, many of the defense systems encoded by phage satellites target multiple phages but not their helper [100], thus increasing the survival of both the host and the helper. Such cooperative strategies are consistent with longstanding coevolution between hitchers, their helpers, and their common host, because they all share, to a certain extent, interest in the survival of the latter. The abovementioned example of mobilizable plasmids providing antibiotic resistance to the bacterial host may also favor helpers indirectly when favoring their common host. Hence, there is extensive potential for shifts towards mutualism in hitcher–helper–host interactions, especially when helpers are not very virulent to the host and hitchers are not very costly to the helpers.

Yet, these mutualistic interactions may be ephemeral. The contribution to host fitness of HGE-encoded accessory traits (e.g., antimicrobial resistance or anti-phage defense), may be positive under certain circumstances (e.g., presence of antimicrobials or phages), and negative under different conditions [102,103]. The mutualistic interactions between hitchers, helpers, and the host thus depend on specific physiological and environmental conditions that may change rapidly. When alliances break the consequences can be brutal, e.g., phages and their satellites will be transferred between cells at the cost of killing the host. We favor the view that such tripartite interactions evolve in a shifting equilibrium between mutualism and antagonism [86]. Understanding the relative frequencies of each type of interaction and the determinants of the shifts will be instrumental to understand the dynamics of these intracellular ecological interactions.

## When and how did hitchers emerge?

Dating the evolution of bacterial hosts is hard because of phylogenetic uncertainty and the lack of fossil record. Dating the origin of MGEs is even harder because they endure rampant recombination, rapid turnover of gene repertoires, and pervasive horizontal gene transfer between bacteria. Available data suggests that some HGEs are very ancient, which is indicative of their resilience and adaptability as successful genetic entities. For example, P4-like satellites are distributed across the order Enterobacterales [12] with some evidence of isolation between the most distant bacterial clades, which might set their origin to hundreds of millions of years ago. This means that they could be older than mammals [104]. Likewise, some relaxases specific of mobilizable plasmids (MOB_P5/HEN_) show distinctive conserved motifs compared to the relaxases of conjugative elements, suggesting they emerged a long time ago [105].

Despite the difficulties in dating their origins, it is clear that hitchers arose many times independently in natural history. This is obviously the case for elements with unrelated types of helpers (phages versus conjugative). It is also the case within each type of HGE and even within families. Conjugative hitchers have paraphyletic or even non-homologous relaxases, the key proteins involved in mobilization by conjugation. Among the 9 relaxase families, 5 harbor a canonical HxH motif (MOB_F_, MOB_Q_, MOB_P_, MOB_V_, MOB_B_) [105,106]. The others are unrelated and likely arose from different enzyme classes: the MOB_H_ family is related to HD-hydrolases [107], the MOB_C_ family is related to restriction endonucleases [108], the MOB_T_ family related to *Rep_trans* rolling-circle replication proteins [109], and the MOB_M_ family is related to tyrosine recombinases [110]. Crucially, the mobilization of hitchers by conjugation through these evolutionary unrelated MOB families suggests they have emerged several times independently [105,106]. Likewise, phylogenetic trees of the capsid genes of cfPICI revealed 3 distinct radiations of these elements [15]. Similarly, HGEs implicated in intra-genomic mobility such as MITES and CALIN emerge regularly in bacterial genomes from transposable elements and integrons, respectively [35,111–113].

**Fig 3:**
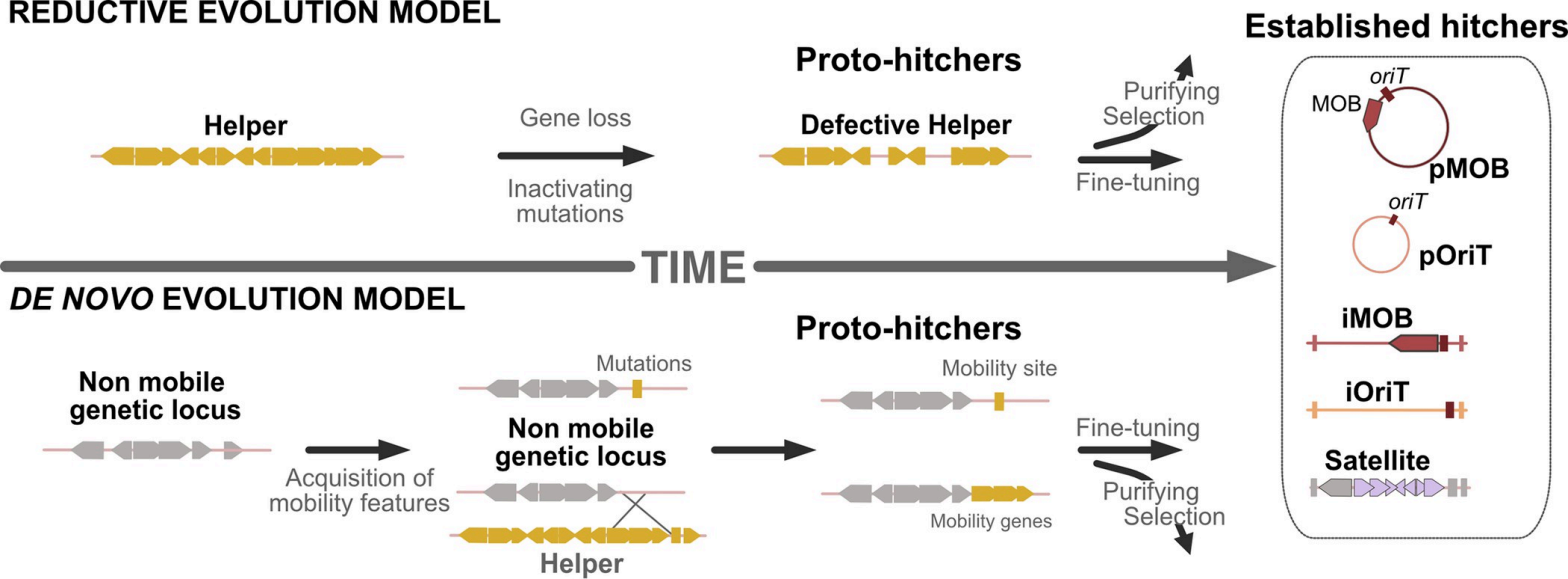
Evolutionary models for the emergence of HGEs. In the reductive evolution model (top), autonomous elements (helpers) evolve towards proto-hitchers by gene losses and/or inactivating mutations. In the de novo evolution model (bottom), non-mobile elements and/or genomic islands evolve towards proto-hitchers by acquisition of mobility genes or DNA motifs. In both scenarios, the proto-hitchers undergo fine-tuning and eventually become established hitchers. pOriT, plasmid carrying *oriT*; *oriT*, origin of transfer, MOB, relaxase.

What are evolutionary processes at the origin of hitchers? We propose 2 alternative models: the reductive evolution model and the de novo evolution model (Fig 3).

Gene loss in a phage or conjugative element may result in a partially defective MGE (proto-hitcher) that is functionally dependent on another element for its transfer between cells. This is the basis of the reductive evolution model for the emergence of HGEs. In this model, the proto-hitcher initially resembles its helper and will progressively diverge from it. Since processes of gene loss occur at high frequency, and can be accompanied by genetic exchanges, they have the potential to create very intermingled evolutionary histories between hitchers and their helpers. This has been most extensively described in mobilizable plasmids which can emerge by the loss of conjugation-related genes. The phylogeny of the most frequent relaxases shows that conjugative elements are ancestral and that gene loss very often gives rise to plasmids with defective conjugation systems that can only be mobilized *in trans* [44,114]. The current paradigm is that MITES and CALIN emerged by gene deletion: loss of the transposase for the former [115] and integrase loss or translocation for the latter [35]. Many bacterial genomes encode defective prophages that could be proto-hitchers [116]. For example, DLP12-like and Rac-like cryptic prophages are found in 74% of *E*. *coli* strains [117]. Detailed analyses of an *E*. *coli* O157:H7 revealed 18 prophages, most of which are inducible and capable of packaging DNA but only 2 are fully functional [118]. Some of these elements have defective structural genes, suggesting that these functions might be provided by other prophages. It remains to be understood how frequently the defective elements can be mobilized by helpers and whether they survive in the long term to become efficient HGEs.

There is reason to believe that many defective elements that work as proto-hitchers are lost in the process. Phylogenies of relaxases reveal many more transitions of conjugative to mobilizable plasmids than the inverse [44], suggesting source-sink evolutionary dynamics where most proto-hitchers are quickly lost. This may be explained by a poor initial adaptation of the MGE to its new role. These proto-hitchers lack many of the characteristics that would favor their efficient mobilization by a helper. Notably, their non-functional mobility systems may be costly for the cell (due to, e.g., production costs or the toxicity of aggregated but non-assembled protein components) and may interfere with the one of the helpers (since they are homologous). Their cost to the cell, when it exists, and their less efficient transfer may result in the frequent extinction of these elements. Some proto-hitchers might also be too related to the helper for their stable maintenance in the population. For example, a defective conjugative plasmid is initially incompatible with its closely related helper due to their similar replication and partition systems. If the proto-hitcher cannot stably co-reside with the helper in the cell, it will quickly become extinct [119]. Accordingly, genome analyses show that plasmids successfully transitioning from conjugative to mobilizable rapidly acquire novel replication initiators [44]. Similar problems may arise when phage satellites emerge from defective prophages and the original element has superinfection exclusion systems that precludes co-infections by the proto-hitcher and its helper [120]. In such cases, the newly formed hitchers might rarely co-reside with the suitable helper, increasing their chances of extinction. A key bottleneck of the reductive evolution pathway may thus be the transition from being a defective helper (proto-hitcher) to become an efficient hitcher.

Alternatively, existing autonomous MGEs may have acquired the ability to be mobilized by other unrelated MGEs. This de novo evolution model predicts that hitchers can emerge from elements unrelated to the helpers. This fits well the available data on some types of satellites, like the P4-like satellites and PLE, which have very few homologs to their helper phages [12,16], and none concerning the proteins responsible for subverting the helpers. Likewise, the relaxases of many mobilizable plasmids are rarely found among conjugative plasmids [105,106]. How could these functions emerge to generate a hitcher? One possibility is that potential proto-hitchers acquired them through genetic exchanges. This is the case of the pOriTs pCERC7 and pBuzz, which have acquired the *oriT* region of the conjugative plasmids R64 and p838B-R, respectively [121,122]. Likewise, PICI satellites have phage-like DNA packaging systems [14], and the capsids of cfPICI satellites form 3 distinct clades within the phage capsid phylogeny suggesting they were independently coopted a long time ago from phages [15]. Such genetic exchanges may be followed by mutations or genetic reassortments resulting in fine-tuning the hijacking functions to their new role in the novel hitcher.

Another possibility is that such functions evolve de novo in mobile genetic elements that were initially non-transmissible between cells. For example, short DNA motifs such as *oriTs* for conjugation or *cos/pac* sites for transduction might emerge by random mutations or recombination events. This process might not be too unlikely given the small size and the low specificity of some of these DNA motifs [77,123]. Recently, it was shown that phages and satellites may transduce plasmids in *S*. *aureus* [47], an indication that packaging signals with sufficient efficiency may arise easily in MGEs. Since MGEs evolve fast and mobility can be under strong selection, relatively inefficient DNA motifs may quickly evolve and improve their ability to mediate the novel hitcher mobility. A mix of mutation and recombination events can also generate novel hitcher-specific genes. For example, MOB_T_ relaxases, encoded by many elements mobilized by conjugation in Firmicutes, are a combination of 2 domains present in other proteins, one related to RCR initiator proteins of the Rep_trans family and another to helix-turn-helix proteins binding DNA [109]. It is also possible, albeit less likely, that new genes encoding hijacking machineries are created de novo, as recently shown for other types of functions [124].

Regardless of the evolutionary pathway leading to the emergence of proto-hitchers, the success of these elements requires further evolution to improve their mobilization and ability to sense and manipulate helpers. In some cases, this has resulted in remarkable evolutionary innovations. For example, Psu and Sid, 2 proteins in P4-like satellites that are involved in manipulating the capsid size of their helpers [22], have no sequence homologs. This occurs in a context where helpers may evolve to avoid the interference of hitchers (if the latter are costly to the former). The ability to be in a transferable state during the self-mobilization of its helper might be one of the most important features for a successful hitcher, especially for those that must excise from the chromosome before transfer. Proto-hitchers may initially be both “mute and blind” regarding when (or if) transfer might occur. How hitchers subsequently acquire and fine tune these traits is a promising path of research to understand their emergence and evolution.

## Less is more?

One intriguing property common to all types of HGEs is that they tend to be much smaller than those of their helpers. For example, conjugative elements have a median size more than 5 times larger than the mobilizable elements [43,89], even if a few exceptions have been described [125]. The genomes of phage satellites are also typically much smaller (6 to 18 kbs) [11,17] than those of their helpers (dsDNA phage genomes are usually more than 25 kb). One may assume that hitchers have smaller genomes than their helpers simply because they do not need to encode mobility functions. Conjugation systems require at least a dozen genes and often many more, and genes encoding viral particles are a substantial fraction of the gene repertoires of temperate phages.

Yet, these reasons do not seem enough to explain the hitchers’ small sizes. There is extensive evidence that conjugation can transfer long replicons, e.g., the historical Hfr strains can conjugate the entire *E*. *coli* chromosome. Furthermore, the size difference between mobilizable and conjugative plasmids (>100 kb) [43] is much larger than the average size of loci encoding the conjugative system [126]. Although some of the mechanisms used by satellites to hijack viral particles do constrain the size of their genomes (e.g., P4 and PLE shrink the capsids of their helpers so that they can only package themselves [22,127]), other satellites do not resize the capsid of their helpers and even package multiple copies of their DNA within the viral particle [17]. The cfPICI satellites even produce their own capsids [15], which could provide them with the possibility of having larger genomes than their helpers (which is not the case [11]). Recent data shows that when one compares old lineages of mobilizable plasmids with recent ones, the former are smaller, suggesting that natural selection favors the streamlining of HGEs [44]. All these observations suggest a pervasive trend for hitchers to be smaller than their helpers even when there are no obvious mechanistic reasons for that. One possible explanation is that larger MGEs express a wider diversity of functions, which renders them more likely to conflict with their hosts and other MGEs. Hence, the streamlining of hitchers’ genomes could decrease carriage costs and favor vertical propagation within bacterial lineages, which would eventually increase the chances that the element co-occurs with a helper.

The small size of hitchers might allow them to better evade defense mechanisms of hosts and other MGEs. For example, small plasmids can more easily escape restriction-modification systems without encoding anti-restriction [68]. Available data suggests that helpers are more specifically targeted by defense systems, hinting that they might be more costly to the cell and other MGEs. For example, some host-encoded defense systems target phage-capsid genes [128], and plasmid-encoded CRISPR-Cas systems target conjugative systems of other conjugative plasmids [129]. The hitchers’ simplicity might thus be the result of a trade-off between autonomy in genetic mobility and size, where the smaller gene repertoire of HGEs increases their chances of persisting by transfer (lower cost to helper), by vertical inheritance (lower cost to bacterial host), or by evasion of bacterial immune defenses.

## Conclusion: A broader view of Hitcher Genetic Elements across the tree of life

The very mechanisms that allow autonomously mobile MGEs—phages and conjugative elements—to spread between cells can be hacked by other MGEs to transfer themselves. Some hitchers may have started as cheaters, i.e., elements that evolve to use the transfer machinery of closely related MGEs. Yet, it is now clear that many hitchers have evolved for a long period of time to become sophisticated streamlined elements specialized in hijacking functions from other MGEs. In this process, they have invented novel machineries to subvert phage packaging or plasmid conjugation, and to defend themselves (and their host) from other MGEs. Many conserved genes in known hitchers remain of function unknown and may be treasure troves of novel biochemical functions, potentially with biotechnological utility.

The recent observation that many MGEs (hitchers) are mobilized by other MGEs (helpers), challenges our understanding of the processes underlying horizontal gene transfer. Hitchers carry traits that influence the complex network of interactions between cells within populations (e.g., bacteriocins, antibiotic resistance). They are also key components of the networks of interactions between MGEs within cells: they modulate the stability, mobilization, and transferability of other MGEs. As such, they have very diverse impacts on the fitness of the bacterial host and of the helper. Whether they are hyper-parasites or hyper-mutualists may depend on the specific triplet host–helper–hitcher, on the accessory traits they carry, and on the circumstances. The last point is essential since it implies these host–helper–hitcher assemblages make niche-dependent alliances that may latter fall apart. These alliances are quite striking regarding defense systems. It was previously pointed out that many, possibly most, “bacterial” defense systems are encoded in MGEs, which means that defense systems are best understood in the context of interactions between MGEs within cells [130]. The protection provided by satellites from some phages is a clear indication of the limits of alliances between hitchers and hosts because the satellites will not protect the bacteria from their helper phages. The abundance of defense systems in hitchers may also result in barriers to horizontal gene transfer between bacteria.

Hitchers have been known for decades but their relevance is only now becoming fully appreciated. Contrary to previous belief, they are not rare defective MGEs on their way to extinction. Instead, hitchers are a category of highly sophisticated mobile genetic elements that is distinctive, often ancient, and very diverse. Our recent ability to identify them has shown that they are very numerous, often outnumbering their helpers. It is not yet clear if this abundance is the result of their selfish spread across communities, of their ability to provide adaptive functions to helpers and hosts, or a mixture of the two. Still, the remarkable functional, structural, and evolutionary parallelisms between very different hitchers, coupled with their abundance across some bacterial clades, suggest that becoming mobilizable by other MGEs can be a successful evolutionary strategy.

Hitchers can also be found in the genomes of eukaryotes and Archaea. MITES are frequent in plants, where they play a key role in promoting genomic plasticity [131,132], and in other eukaryotic organisms [133]. In Archaea, ca. 20% of the genomes contains MITEs [32]. Many eukaryotic viruses are satellites of autonomous viruses. One well-described example is the hepatitis D virus, a small, “defective” RNA virus. It is the smallest known virus that infects humans and causes the most severe form of viral hepatitis. The hepatitis D virus does not encode the surface antigens that allow it to infect human cells, but instead relies on those encoded by the hepatitis B virus [134]. Since the generation of defective interfering particles is common during viral infection [135,136], many eukaryotic virus-like hitchers emerge, at least temporarily, through reductive evolution (Fig 3). Eukaryotes are also infected by virophages, elements that encode their own viral structural genes but require the viral particle factory of giant viruses for replication [137]. Archaeal viruses also have their satellites [138]. While conjugation is unknown in eukaryotes, it is present in Archaea and hitchers mobilizable by conjugation might exist. Their identification has lagged behind because we still ignore proteins carrying the function of relaxases in Archaea [139]. It is thus likely that many of the challenges and outlooks that we describe here apply to HGEs in other domains of life, many of which may remain undiscovered. Becoming mobilizable, instead of autonomously mobile, might have been frequent, perhaps even inevitable, in the evolution of life.

## Abbreviations

cfPICI: capsid-forming PICI
HGE: Hitcher Genetic Element
ICE: integrative conjugative element
IME: integrative mobilizable element
MGE: mobile genetic element
MITE: miniature inverted repeat transposable element
MPF: mating pair formation
PICI: phage-inducible chromosomal islands
pCONJ: conjugative plasmid
PLE: PICI-like element
